# Immunosuppressive IDO in Cancer: Mechanisms of Action, Animal Models, and Targeting Strategies

**DOI:** 10.3389/fimmu.2020.01185

**Published:** 2020-06-16

**Authors:** Lijie Zhai, April Bell, Erik Ladomersky, Kristen L. Lauing, Lakshmi Bollu, Jeffrey A. Sosman, Bin Zhang, Jennifer D. Wu, Stephen D. Miller, Joshua J. Meeks, Rimas V. Lukas, Eugene Wyatt, Lynn Doglio, Gary E. Schiltz, Robert H. McCusker, Derek A. Wainwright

**Affiliations:** ^1^Department of Neurological Surgery, Feinberg School of Medicine, Northwestern University, Chicago, IL, United States; ^2^Division of Hematology and Oncology, Department of Medicine, Feinberg School of Medicine, Northwestern University, Chicago, IL, United States; ^3^Robert H. Lurie Comprehensive Cancer Center of Northwestern University, Chicago, IL, United States; ^4^Department of Microbiology-Immunology, Feinberg School of Medicine, Northwestern University, Chicago, IL, United States; ^5^Department of Urology, Feinberg School of Medicine, Northwestern University, Chicago, IL, United States; ^6^Department of Dermatology, Feinberg School of Medicine, Northwestern University, Chicago, IL, United States; ^7^Department of Biochemistry and Molecular Genetics, Feinberg School of Medicine, Northwestern University, Chicago, IL, United States; ^8^Division of Neuro-Oncology, Department of Neurology, Feinberg School of Medicine, Northwestern University, Chicago, IL, United States; ^9^Department of Pharmacology, Feinberg School of Medicine, Northwestern University, Chicago, IL, United States; ^10^Transgenic and Targeted Mutagenesis Laboratory, Feinberg School of Medicine, Northwestern University, Chicago, IL, United States; ^11^Center for Molecular Innovation and Drug Discovery, Feinberg School of Medicine, Northwestern University, Chicago, IL, United States; ^12^Department of Animal Sciences, University of Illinois at Urbana-Champaign, Urbana, IL, United States

**Keywords:** aging, immunotherapy, glioblastoma, tryptophan, immunosuppression, Treg, IDO1, kynurenine

## Abstract

Indoleamine 2, 3-dioxygenase 1 (IDO; IDO1; INDO) is a rate-limiting enzyme that metabolizes the essential amino acid, tryptophan, into downstream kynurenines. Canonically, the metabolic depletion of tryptophan and/or the accumulation of kynurenine is the mechanism that defines how immunosuppressive IDO inhibits immune cell effector functions and/or facilitates T cell death. Non-canonically, IDO also suppresses immunity through non-enzymic effects. Since IDO targeting compounds predominantly aim to inhibit metabolic activity as evidenced across the numerous clinical trials currently evaluating safety/efficacy in patients with cancer, in addition to the recent disappointment of IDO enzyme inhibitor therapy during the phase III ECHO-301 trial, the issue of IDO non-enzyme effects have come to the forefront of mechanistic and therapeutic consideration(s). Here, we review enzyme-dependent and -independent IDO-mediated immunosuppression as it primarily relates to glioblastoma (GBM); the most common and aggressive primary brain tumor in adults. Our group's recent discovery that IDO levels increase in the brain parenchyma during advanced age and regardless of whether GBM is present, highlights an immunosuppressive synergy between aging-increased IDO activity in cells of the central nervous system that reside outside of the brain tumor but collaborate with GBM cell IDO activity inside of the tumor. Because of their potential value for the *in vivo* study of IDO, we also review current transgenic animal modeling systems while highlighting three new constructs recently created by our group. This work converges on the central premise that maximal immunotherapeutic efficacy in subjects with advanced cancer requires both IDO enzyme- and non-enzyme-neutralization, which is not adequately addressed by available IDO-targeting pharmacologic approaches at this time.

## Introduction

**Figure 1:**
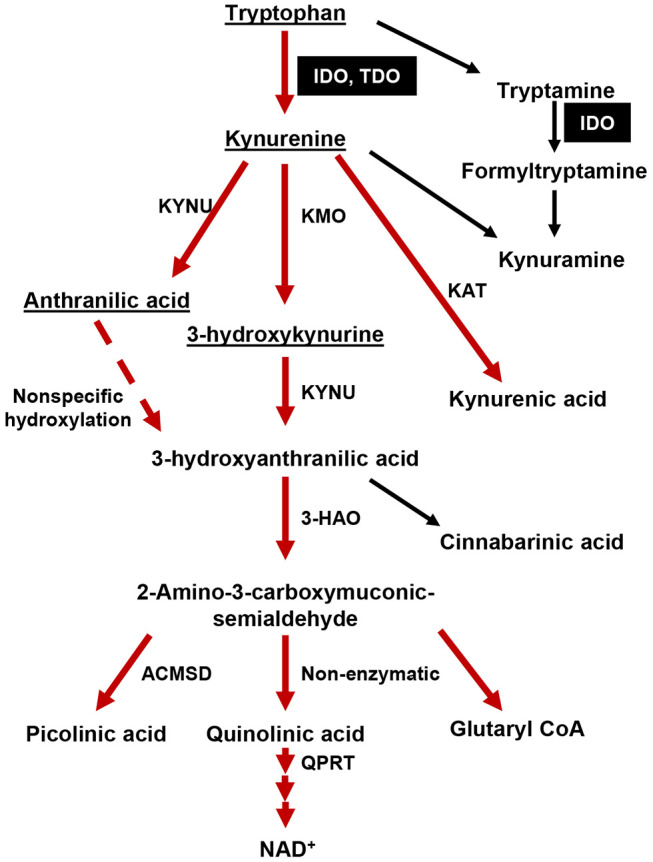
The tryptophan (Trp)→ kynurenine (Kyn) metabolic pathway. The majority of dietary tryptophan (95%) is metabolized via the TrpKyn pathway (red arrows). A minor pathway that converts tryptamine into kynuramine is also included. Metabolites capable of crossing the blood brain barrier (BBB) are underlined. IDO and TDO are highlighted in black boxes. KMO, kynurenine 3-monooxygenase; KYNU, kynureninase; KAT, kynurenine aminotransferase (I, II, III); 3-HAO, 3-hydroxyanthranilate 3,4-dioxygenase; ACMSD: 2-amino-3-carboxymuconate semialdehyde carboxylase; QPRT, quinolinic-acid phosphoribosyl transferase.

Tumors arise from cell-intrinsic pro-growth mutations, the catastrophic dysfunction of host immune defense, and/or the evolution of tumor cell-intrinsic mechanisms that facilitate host immune system evasion. Amino acid metabolism is a fundamental biological event intrinsic to all living organisms that has long been recognized to play a crucial regulatory checkpoint, resulting in the sculpting of cellular responses during pathophysiological conditions such as infectious disease, autoimmunity, neurodegeneration, psychiatric conditions, as well as cancer (1). Over the past two decades, the kynurenine (Kyn) pathway of tryptophan (Trp) metabolism (Figure 1) has been intensely studied throughout both preclinical and clinical therapeutic settings (2). The majority of this work has focused on the two primary rate-limiting enzymes that catalyze the first step of Trp metabolism: indoleamine 2, 3-dioxygenase 1 (IDO) and tryptophan dioxygenase (TDO). The central dogma underlying the immunosuppressive role for these enzymes is associated with their canonical Trp catabolic properties: IDO and/or TDO-mediated depletion of Trp and/or the accumulation of Kyn, which is associated with the suppression of immune effector cells and the upregulation, activation, and/or induction of tolerogenic immune cells (3, 4). Although a plethora of *in vitro*-motivated investigation has generated compelling evidence confirming IDO enzyme activity as a source of therapeutic value, data challenging the Trp depletion dogma as the only mechanism by which IDO suppresses the immune response began emerging almost a decade ago. Furthermore, the phase III Keynote-252/ECHO-301 clinical trial that evaluated combination treatment with the potent IDO enzyme inhibitor, epacadostat, and pembrolizumab [anti-programmed cell death 1 (PD-1)] in patients with unresectable or metastatic melanoma, failed to meet its primary end point as compared to individuals treated with pembrolizumab as a monotherapy (5). Despite certain variables that include objective *in vivo* efficacy and the underlying rationale for this combination of therapy which may have contributed to its clinical failure (6), a careful consideration for IDO-targeting approaches is warranted. Additional conflicting results that describe the role of IDO across different cancers and the cell types expressing the immunosuppressive mediator highlight the various underlying mechanisms that are context-dependent, multi-dimensional, and temporally-sensitive.

Here, we summarize current knowledge of IDO-mediated immunomodulation with a focus on how it affects the anti-cancer immune response. Potential mechanism(s) that reshape and/or revise current IDO dogma as it relates to the cancer immunity cycle are also explored. Recent advances in our understanding of IDO expression changes during aging and the potential contribution of these effects on suppressing immunosurveillance mechanisms during cancer cell initiation and/or tumor cell outgrowth are also discussed. Finally, the diametrically-opposed relationship between intratumoral IDO levels and overall survival among different types of cancer patients will provide a unique perspective on how cancer immunity dogma is not universally applicable.

### IDO, Trp Metabolism and Its Association With Suppressing the Anti-cancer Immune Response

Less than 1% of dietary Trp is used for protein synthesis under physiological conditions while the remainder is degraded through decarboxylation, transamination, hydroxylation, or oxidation (7), which leads to the generation of physiologically active compounds including neuroactive tryptamine, neuroprotective melatonin, and/or immunomodulatory kynurenines. IDO and TDO catalyze the rate-limiting cleavage of the Trp indole ring 2, 3-double bond and incorporate molecular oxygen. The product of this reaction is *N*-formylkynurenine (NFK) that is rapidly converted into Kyn by formamidase. The latter catabolite is further transformed into downstream kynurenine-like intermediates, including 3-hydroxy-_L_-kynurenine (3-HK), 3-hydroxyanthranilate (3-HAA) and quinolinic acid (Quin), which also affect the immune response (8). The ultimate product of Kyn pathway metabolism is nicotinamide adenine dinucleotide (NAD^+^), a critical enzyme co-factor involved in a variety of fundamental biological events including acting as an electron transfer factor during glycolysis and oxidative phosphorylation, as well as being consumed by covalent modification of proteins and signaling enzymes. While IDO and TDO share similar functions as a mediator of Trp degradation, their biochemical properties are quite distinct. Monomeric IDO can act on a broad range of substrates that include _L_-Trp, _D_-Trp, tryptamine, 5-hydroxytryptophan and 5-hydroxytryptamine. In contrast, homotetrameric TDO is enantiomer specific and only metabolizes _L_-Trp (9). The affinity of IDO for Trp and Trp-derivative substrates is highly variable such that binding to _D_-Trp is low as compared to _L_-Trp, with a *K*_m_ of human IDO for the _D_-isomer that is 200-fold higher than that for the _L_-isomer (10, 11). In contrast to IDO and TDO, the third Trp catabolic enzyme, IDO2, possesses a negligible level of intrinsic enzymic potential. The *K*_m_ of human IDO and IDO2 for _L_-Trp is 20.90 ± 3.95 μmol/L and 6,809 ± 917 μmol/L, respectively (12) supporting the hypothesis that suggests IDO2 contributes a minimal role to overall Trp metabolism in humans. The lack of change in absolute Kyn among cell lines expressing high IDO2 cDNA levels, as well as the negligible change in systemic Kyn levels in mice with constitutional IDO2 deficiency (13–15), further support this hypothesis.

In addition to the distinct metabolic properties associated with Trp metabolism, the factors regulating IDO gene transcription and translation are different as compared to those that regulate TDO and IDO2 expression. Pro-inflammatory mediators tend to be the strongest agonists of IDO transcription that include the prototypical antitumor-associated T cell effector cytokine, interferon-gamma (IFN-γ), as well as the toll-like receptor 9 (TLR9) and TLR4 agonists, CpG DNA and lipopolysaccharide (LPS), respectively (16–19). Tumor necrosis factor-alpha (TNF-α), interleukin-6 (IL-6) and IL-1β also synergize with one another to facilitate the upregulation of IDO expression. Other modulators include soluble glucocorticoid-induced TNFR-related protein (GITR), prostaglandin E2, the oncogene c-Kit, as well as the tumor suppressor, Bin1 (20). The soluble fusion protein, cytotoxic T lymphocyte-associated protein 4-immunoglobulin (CTLA-4-Ig) induces IDO expression in dendritic cells (DCs) through the interaction of co-stimulatory receptors, CD80 (B7.1) and CD86 (B7.2) (21). Recent work further confirms that Wnt5α is another enhancer of IDO activity during β-catenin signaling in DCs (22).

Constitutive IDO expression can be found in healthy human subjects but is highly restricted to a select number of tissues and cells including mature DCs in secondary lymphoid organs, epithelial cells inside the female genital tract, as well as endothelial cells of term placenta and lung parenchyma (23). It should be noted that this superficial analysis does not capture the potential expression changes that occur across age groups as we previously found in the normal human brain parenchyma during advanced age (24). While some human tumor cell types constitutively express IDO, its presence is normal absent in human and mouse glioblastoma cells but is rapidly induced upon treatment with human and mouse IFNγ (25–29). Constitutive TDO expression is also expressed among different organs of healthy human subjects including bone marrow, muscle, gastrointestinal tract, kidney and urinary bladder, as well as inside the brain, with the highest levels in liver hepatocytes (30). TDO expression levels are regulated in-part by systemic levels of _L_-Trp and corticosteroids (31, 32). Notably, common corticosteroid-like treatments such as dexamethasone rapidly increases TDO levels across multiple cell types. Similar to IDO, TDO is expressed by a bevy of different tumor types including glioblastoma, melanoma, ovarian carcinoma, hepatic carcinoma, breast cancer, non-small-cell lung cancer, lymphoid and myeloid cancers, renal cell carcinoma, and bladder cancer, with high expression levels associated with a more rapid rate of tumor progression and/or decreased patient survival (33–36). IDO2 mRNA is constitutively expressed in the human liver, brain, thyroid, placenta, endometrium, and testis, and is induced in antigen-presenting cells (APCs) and B cells (37). However, the role of IDO2 in affecting the anti-cancer immune response is less clear with the analysis of 129 human tumor samples and 25 human tumor cell lines showing no detectable full-length IDO2 mRNA transcript (36).

The primary dogma establishing a relationship between IDO, TDO, IDO2 and immunosuppression is associated with their individual and/or collective contribution toward the metabolism of Trp. The premise for this dogma is based on the Trp starvation theory that postulates the near absolute depletion of Trp, at or below <1 μM, facilitates the accumulation of uncharged tRNAs which then activate the general control non de-repressible 2 (GCN2) kinase pathway and the dysfunction of T cells (38). *In vitro* studies support the hypothesis that Trp depletion inhibits the master metabolic regulator mammalian target of rapamycin (mTOR) and protein kinase C (PKC-Θ) in cancer cells, which consequently enhances autophagy and T_reg_ development, respectively (39). Trp degradation may also suppress immune cell activities through the formation of Kyn and downstream derivative metabolites. *In vitro* and further requiring co-treatment with transforming growth factor-beta (TGF-β), Kyn facilitates the induction of FoxP3 in naïve CD4^+^ T cells by activating the aryl hydrocarbon receptor (AhR) (40), a ligand-activated transcription factor that exerts potent effects on immune cells (41) and is involved in the differentiation of inducible Tregs (42, 43). The downstream pathway Kyn metabolites including kynurenic acid (KA) (44), xanthurenic acid (XA) (35), and cinnabarinic acid (CA) (45) interact with AhR and may also play a role in modulating the immune response. In striking contrast, Trp catabolites have been demonstrated to also induce CD4^+^ T cell apoptosis. Terness et al. found that Kyn, 3-HK, and 3-HAA suppress T cell proliferation coincident with the induction of apoptosis (46). This finding was independently confirmed by Fallarino et al. (47) demonstrating *in vitro* that, Kyns induce the selective apoptosis of murine thymocytes and Th1-, but not Th2-cells. This immunoregulatory role of Kyns on different lymphocyte subsets may be important for maintaining peripheral lymphocyte homeostasis and for minimizing the accumulation of autoreactive and/or inflammatory lymphocytes. However, the exact molecular mechanism of Kyn-mediated selective apoptosis on T cell subsets remains unknown.

Despite a literature replete with previous work supporting the hypothesis that IDO-mediated Trp metabolism enhances suppression of the anti-cancer immune response, it's important to acknowledge that the predominant source for which these studies are based upon is derived from *in vitro* cell culture. Since Trp is an essential amino acid and not readily generated by mammalian cells, dietary consumption is critical for host survival and *in vivo* replenishment. It is therefore important to consider that the Trp concentrations required to inhibit T cell proliferation *in vitro* have been shown to be below 0.5–1 μM (38). Whether this cell culture condition can be transposed upon physiological conditions *in vivo* is controversial since human plasma Trp levels normally range between 50 and 100 μM. A number of conflicting results on this topic have recently arisen and was highlighted by Frumento et al. during their failure to observe an inhibition of T lymphocyte proliferation when the *in vitro* cell culture media was absent for Trp (48). Although it is arguable that high expression of IDO (or TDO) in tumor cells might lead to extremely low Trp levels within the tumor microenvironment, it's very difficult to fully validate this argument *in vivo*. Using the matrix-assisted laser desorption/ionization-time of flight mass spectrometry (MALDI-TOF MS) imaging technique, Sonner et al. (49) recently reported on TDO-mediated immunosuppression in experimental murine melanoma and demonstrated that, even in B16 melanoma highly expressing TDO by virtue of a cDNA-expressing plasmid, the intratumoral Trp levels were maintained at a similar level as compared to those inside unmodified B16 melanoma; thus suggesting that *in vivo* Trp levels are very stable even when Trp metabolism is maintained at an abnormally high rate.

Another hypothetical explanation regarding Trp depletion relies on the alteration of Trp transporter activity. This theory posits that even while there is no dramatic decrease of Trp in the peripheral blood circulation, alteration of the Trp transporter activity may cause intracellular Trp depletion which further triggers downstream immunosuppressive signals. In addition to the well-known non-specific low affinity Trp transporter (system L) (50), two Trp specific transporters with high affinity have been reported to be expressed on monocyte-derived macrophages and tumor cell lines (51, 52). Unfortunately, neither of these novel Trp transporters has been successfully cloned. Comprehensive functional analyses of the Trp transporters expressed by different immune cells and/or tumor cells *in vitro* and *in vivo* remain largely unexplored.

Intriguingly, T cell specific GCN2 knockout mice have similar anti-tumor effect against syngeneic B16 melanoma as compared to their wild type counterparts (49). Moreover, despite the elegant *in vitro* work by Mezrich et al. reporting that the 50 μM Kyn treatment activates AhR in naïve T cells and subsequently induces FoxP3 expression (40), human serological Kyn levels are normally within the range of 2–3 μM in both healthy individuals and in patients with inflammatory conditions (53–57). Theoretically, local tissue Kyn levels might transiently increase when IDO expression is highly expressed (58), but more precise *in vivo* detection and quantification techniques remain critically unavailable. Moreover, whether Kyn possesses an inducible or inhibitory effect on DC-mediated T_reg_ differentiation is also debatable. Somewhat conflicting with the report by Mezrich et al., Nguyen et al. reported that the addition of Kyn in a DC-T cell co-culture system led to the inhibition of naive T cell differentiation into FoxP3^+^ Tregs (59). This latter observation supports the hypothesis that the Kyn-AhR mechanism is likely not responsible for the direct generation of Tregs in solid tumors; especially since the infiltrating component of tumors tend to primarily consist of thymus-derived natural T_reg_ (60, 61) that fail to express Ahr (62). It is also notable that other studies indicate the potential role of Kyn on T_reg_ activation as evidenced by the Munn and Mellor group who showed IDO-expressing plasmacytoid DCs in tumor draining lymph nodes preferentially activate pre-existing T_reg_ through the PD-1-PTEN pathway which was inhibited by the IDO pathway non-enzyme inhibitor, 1-methyl-_D_-tryptophan (1-MT) (63, 64). In addition to the sometimes conflicting outcomes discussed above, results from studies suggesting that Kyns directly suppress the actions of effector T cells have also been revisited due to the high doses evaluated during those studies, which ranged from 100 to 1,000 μM (46, 47). The panoply of different observations across various modeling systems highlight the complexity of IDO immunobiology that, may be conveying artifactual effects during *in vitro* study and thereby supports the re-evaluation of previous interpretations with the use of new physiologically-relevant *in vivo* modeling systems.

### Pleiotropic IDO Effects Reflect Multiple Functions, Diverse Cellular Origins, and Varying Kinetics

Beyond the restricted tissues that constitutively express IDO is the induction of its expression in a variety of cell types under normal and pathological conditions. This includes the changes of IDO expression during progressive aging (24), as well as in the setting of malignancy whereby levels change in response to T cell infiltration (65). Further complicating IDO functionality has been highlighted by immunohistochemical IDO detection across 15 different human cancer types that found protein localization in myeloid cells, endothelial cells, tumor cells, or a combination of those origins (23). The select pattern of intratumoral IDO expression raises critical questions including: (i) does IDO perform the same role among different cell types; (ii) how do these cells coordinately contribute to tumor growth and/or suppression of anti-cancer immunity; (iii) what are the kinetics of endogenous IDO expression and its relationship to enzyme activity?

A peculiar trait of tumor cells expressing high IDO expression is the resultant effect on an overall slower rate of growth as compared to cells with lower intrinsic IDO levels (66). A derivative and competing hypothesis arising from this observation suggests that IDO functions as a tumor suppressor. This was corroborated with findings of higher intratumoral IDO expression positively associated with longer survival in renal cell carcinoma-, hepatocellular carcinoma-, and melanoma-patients (3, 67–69). It should also be considered, however, that higher levels of T cell infiltration are often associated with higher intratumoral IDO levels and this relationship has an established survival improvement in human subjects diagnosed with select malignancies (3, 70). Therefore, the higher intratumoral IDO levels are likely associative and may not have any effect on tumor growth itself. Regardless and in contrast to the alleged beneficial effects of slower growth, higher IDO expression was associated with an improved motility of lung cancer cells that enhanced their metastatic formation in the brain, liver, and bone (71), whereas IDO deficiency decreased metastasic burden and improved the survival of subjects with breast carcinoma-derived pulmonary metastases (72, 73). Intratumoral IDO expression also correlated with the frequency of liver metastases in colorectal cancer (74), distant metastases in hepatocellular cancer (69), and nodal metastases in endometrial carcinoma (75). Among human cancer cells, IDO activity has been implicated in improving DNA repair and mediating the resistance to treatments such as the poly-ADP-ribosyltransferase (PARP) inhibitor, olaparib, γ-radiation, and the chemotherapeutic agent, cisplatin, through generation of NAD^+^ (76). It was reported that impaired Trp metabolism resulted in the inhibition of *de novo* NAD^+^ synthesis, which led to hepatic tumorigenesis through DNA damage (77), further supporting the previous finding that NAD^+^ serves as the only endogenous substrate of PARPs to facilitate the removal of oxidative DNA damage. However, it's important to note that IDO is not normally expressed in the liver, whereas, TDO is constitutively and highly expressed. Therefore, if either or both of these factors are associated with the impaired Trp metabolism, it may be associated with pathological injury and not necessarily the normal expression of those rate-limiting enzymes.

While IDO expression by tumor and immune cells receives the predominant attention, the immunosuppressive mediator is also expressed by endothelial cells (23). In healthy tissues, IDO is expressed by endothelial cells in a large proportion of placental and pulmonary blood vessels, as well as by a minority of blood vessels in select other organs. Intratumorally, vascular IDO expression is frequent among renal cell carcinoma, non-small lung carcinoma, endometrial carcinoma, and melanoma. Endothelial IDO appears to modulate vascular tone through several different signaling pathways including Kyn-mediated activation of soluble guanylate cyclase, adenylate cyclase, and voltage-dependent K^+^ channels (78, 79). The Kyn metabolite, xanthurenic acid, has been shown to possess greater potency as compared to _L_-Kyn in causing blood vessel relaxation that is dependent on nitric oxide (80). Stanley *et al*. found that in the presence of H_2_O_2_, IDO catalyzes Trp oxidation using singlet molecular oxygen (^1^O_2_), leading to the vasodilation product *cis*-WOOH (81). It's important to note that these experiments were conducted while utilizing high concentrations of the Trp catabolites between 300 μM to 1.5 mM *in vitro*. It's therefore unclear as to whether the vasodilating compounds attain a similar local level inside a solid tumor *in vivo*. However, other observations support the vaso-relaxing effects of endothelial IDO including a model of sepsis-induced hypotension (82). Intrinsic to the IDO expressed by endothelial cells there may also be a role for an effect on modulating tumor neovascularization (83), though the exact molecular mechanism explaining has yet to be elucidated (84).

Data suggesting that IDO possesses non-metabolic functions independent from those associated with its enzyme activity began emerging ~10 years ago. Pallotta *et al*. was the first to report that the *in vitro* treatment of TGF-β in cultures of mouse plasmacytoid DCs (pDCs) leads to the phosphorylation of IDO immunoreceptor tyrosine-based inhibitory motifs (ITIMs), which subsequently recruits and activates the tyrosine phosphatases, SHP-1 and SHP-2, as well as inositol polyphosphatase (SHIP) (85). Activation of the pDC TGF-β-IDO-SHP axis enabled an autocrine loop through the induction of non-canonical NF-κB signaling, which further enhanced intra-pDC IDO expression levels. Treatment of the pDCs with 1-methyl-_D, L_-tryptophan confirmed that the immunosuppressive IDO signaling mechanism was faithfully independent of its association with mediating Trp metabolism. However, it's notable that the 4 μM dose of 1-MT used in this study was lower than the EC_50_ of 1-MT (13). Future validation and replication of these results while utilizing a more potent IDO enzyme inhibitor will allow for confidence and verification of non-enzymic IDO functions. Our group found similar observations of IDO mediating both enzyme and non-enzyme effects that depend on context. Using a syngeneic brain tumor model, we previously demonstrated that the shRNA knockdown of GBM cell IDO leads to the suppression of intratumoral Treg accumulation and was associated with a significant improvement in long-term animal subject survival (86) independent of GBM cell IDO metabolism (29). We also showed that the forced expression of GBM cell IDO cDNA (IDO-O/E) enhances Treg recruitment even when animal subjects are treated with a potent blood brain barrier-penetrating pharmacologic IDO enzyme inhibitor (87). We further confirmed that while the IDO enzyme inhibitor significantly decreased intratumoral Kyn levels in GBM IDO-O/E *in vivo*, the reduction was not associated with decreased intratumoral Treg accumulation. Taken together, these observations support the hypothesis that new IDO-targeting approaches aimed at simultaneously reversing enzyme and non-enzymic activities will enhance the effectiveness of future cancer immunotherapy efforts.

### IDO and Its Relationship to Host Age

Considering the suppressive role of the IDO/TDO axis in immunoregulation, as well as the changes in immunological status system during progressive aging, it's not surprising that several studies have investigated the relationship between the TrpKyn pathway as it relates to aging-dependent disease. The serological Kyn/Trp ratio is often used an indicator of IDO enzyme activity and progressively increases during normal aging in humans (88, 89). This increase has been associated with enhanced frailty in human subjects >65 years of age and predicts an increased mortality rate of individuals in their nineties (90). Additionally, meta-analysis of age-related gene expression changes in the peripheral blood of adult individuals identified the enzyme kynureninase (KYNU, Figure 1) as one of the most differentially expressed genes (91). In a large population screening analysis that included 7,074 human subjects that focused on studying bone mineralization, Apalset et al. reported that the serological Kyn/Trp ratio negatively correlated with bone mineral density in the 71–74 year old age group as compared to younger individuals that were 46–49 years of age (92). More recently, Ocampo et al. summarized the results from previous animal studies of brain diseases and the interaction with Kyns during aging that indicate a strong correlation between Kyn pathway metabolites across different rodent ages (93). They found an intriguing observation that while TDO and IDO expression decrease in the liver and kidney during progressive aging, TDO and IDO were dichotomized in the rat brain during advanced age such that TDO decreased and IDO increased in overall expression as compared to young animal subjects. Similarly, our group previously demonstrated that there is an ~400 fold increase in IDO mRNA expression in the normal naïve brain of 72–74 week old C57BL/6 WT mice as compared to 6–8 week old subjects (94). Our most recent study evaluating the interactions between normal human aging and its relationship to brain cancer incidence and mortality also found a significant increase in IDO mRNA expression in the normal human brain of individuals aged 60–69 years of age as compared to younger human subjects (24). The increased human brain IDO expression was associated with a maximal T_reg_/CD8^+^ cytolytic T cell ratio in the peripheral blood of the 60–69 year old age group confirming a simultaneous increase of systemic- and local-immune suppression (24). To address these observations, we are currently in the process of determining as to whether the advanced age-dependent increase of central nervous system IDO expression directly increases the peripheral T_reg_/CD8^+^ cytolytic T cell ratio and how this relationship affects the incidence and mortality rate, as well as responsiveness to immunotherapy of GBM.

Understanding how aging-dependent molecular mechanisms affect IDO enzyme and non-enzymic activity is in its infancy. Refaey et al. reported that 22-month old mice have significantly higher serum N-formylkynurenine as compared to younger counterparts at 4 and 13 months of age, suggesting a potential role for Kyn pathway metabolism in aging-dependent bone loss (95). Strikingly, either the *in vitro* addition of Kyn to bone marrow-derived mesenchymal stromal cells (BMSCs), dietary supplementation of Kyn, or a direct administration of Kyn into the peritoneum suppressed the formation of new bone; possibly by decreasing osteoblast formation, failed recruitment capability and/or loss of effector functions. It should be noted, however, that neither age-matched IDOKO nor TDOKO mice were used in the study and it's therefore inconclusive as to the primary source of aging-dependent changes in Kyn levels. Independently, Minhas et al. demonstrated that the catabolic enzyme converting quinolinic acid into NAD^+^, quinolate phosphoribosyltransferase (QPRT), significantly declines in aged human monocyte-derived macrophages (MDMs) from individuals ≥65 years old as compared to MDMs isolated from individuals ≤ 35 years of age (96). Loss of QPRT subsequently resulted in decreased *de novo* NAD^+^ synthesis and was associated with an enhanced pro-inflammatory status and lower phagocytic ability of MDMs. It's worth noting that in addition to the enzymes and metabolites of the Kyn pathway, changes in AhR activity and/or expression have also been implicated during aging (97). However, the direct relationship between IDO, TDO, Kyn, and AhR has not been comprehensively established during a combined investigation of aging and in a specific pathophysiological setting.

Based on the relationship between increased incidence of patients with cancer and advanced age (98), it may be surprising that few studies have investigated IDO and/or TDO changes in subjects with cancer and across the health-/life-span. Adult glioblastoma is an age-related disease with a median age of diagnosis at 65 years old (99). To explore the mechanistic underpinnings between advanced age and its effects on the GBM immune response, we previously compared the survival rate of young 6–8 week- and adult 72–74 week-old mice intracranially-engrafted with the syngeneic GL261 glioma cell line. The young mice survived slightly longer than the older subjects with a median overall survival (mOS) of 27.5 days and 21.5 days, respectively (*p* = 0.0292) (94). Intriguingly, advanced age was associated with a large increase of IDO expression in the contralateral brain without tumor and as compared to young mice, whereas there was no such difference within the tumor itself. These data suggest that the majority of brain parenchymal cells expressing increased IDO during advanced age do not migrate into the GBM. Intriguingly, serological, and intra-GBM Trp and Kyn levels showed no differences between young and adult subjects despite the increased IDO expression in the older brain (94). We also studied the effects of advanced age on immunotherapeutic efficacy in adult mice at 72 weeks of age, which is closer to the analogous time frame of a human GBM patient diagnosis as compared to the standard 6–12 week old mouse. When the young and adult mice underwent therapy (87) with whole brain radiotherapy, anti-PD-1 mAb and pharmacological IDO enzyme inhibitor, older mice showed higher IDO mRNA in the non-tumor brain tissues as compared to younger subjects. Functionally, the triple combination therapy resulted in a median survival of 31.5 days in the older subjects, which was significantly decreased as compared to the younger 8-week-old mice with a survival of 40.5 days (*p* < 0.001), suggesting that the increased brain IDO expression during advanced age has a directly negative effect on immunotherapeutic efficacy in subjects with GBM.

### Mouse Models for Studying IDO

Among preclinical studies, transgenic mouse models have demonstrated their substantial importance to understanding the function, effects and mechanism of IDO in a physiologically-relevant environment. The first IDO knockout (IDOKO) mouse strain originated from the Munn and Mellor group whereby exons 3–5 of IDO were replaced with a β-gal and neomycin cassette (100, 101). Surprisingly, the homozygous IDOKO mice are viable, fertile and possess a phenotypically normal immune system without any obvious graft vs. host-like symptoms, suggesting that IDO-mediated immune suppression is dispensable for the normal maintenance of central and peripheral tolerance to self-antigens. Given the functional similarity between TDO and IDO for mediating Trp catabolism, it's possible that TDO plays a compensatory role in the absence of IDO. However, it's notable that Kyn levels are significantly decreased in naïve IDOKO mice, so if TDO compensates, there appears to be a limit to those potential effects (29). The expression and function of TDO has yet to be investigated in IDOKO mice with cancer. Though IDO expression is not required for immune tolerance under homeostatic conditions, it plays a critical role during acquisition of tumor-immune tolerance as indicated by studies demonstrating that IDOKO mice have increased tumor infiltration of effector T lymphocytes, decreased immunosuppressive immune cells, as well as increased survival (102–105), implicating non-redundant functions between IDO and TDO in the various cancer settings.

**Figure 2:**
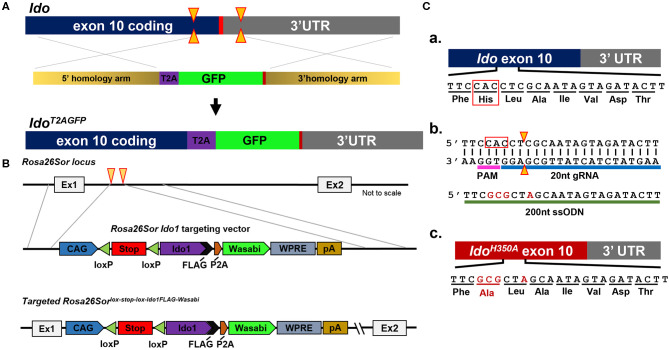
Schema utilized to generate IDO-targeted transgenic mouse strains. **(A)** Generation of the *Ido1*^T2AGFP^ mouse line. CRISPR/Cas9 technology was employed in murine embryonic stem (ES) cells. Two separate guide RNAs directed Cas9 nuclease cleavage of the *Ido1* locus, creating double strand DNA breaks (yellow arrow) before and after the stop codon (red bar). The CRISPR ES cell reaction included a plasmid repair template, which encoded the T2A and GFP elements flanked by 5′ and 3′ homology arms of 700nt (tan bars). The T2A and GFP elements were introduced into the endogenous *Ido1* locus before the stop codon *via* homology directed repair (HDR). **(B)** Introduction of a *Cre*-dependent *Ido1* expression construct into the *Rosa26Sor* locus by CRISPR/Cas9 technology. Two separate guide RNAs directed Cas9 nuclease cleavage at the *Rosa26Sor* locus, creating double strand DNA breaks (yellow arrows). A plasmid repair temple (*Rosa26Sor Ido1* targeting vector) was included in the reaction and introduced *via* HDR. The edited locus encodes the CAG promoter driven, *Cre*-dependent conditional expression of a bi-cistronic *Ido1* construct. The *Ido1* transcript includes a C-terminal FLAG-tag, followed by a P2A element and expression of the Wasabi fluorophore. It also included a WPRE element and the bovine growth hormone polyadenylation (pA) signal. The construct is flanked by 800 bp 5′ and 750 bp 3′ homology arms (gray lines). **(C)** Generation of the *Ido1*^H350A^ mouse line. (a) Schematic showing the last coding exon of the *Ido1* gene, exon 10. The highlighted region encodes a histidine residue at position 350 (red box) that is essential for mouse IDO enzyme activity; (b) CRISPR/Cas9 technology generated a precise double strand DNA break in the *Ido1* exon 10 locus in fertilized mouse embryos (yellow triangle). A 200nt single stranded oligodeoxynucleotide repair template was included in the reaction (green bar). The ssODN introduced point mutations (red letters) into the endogenous *Ido1* locus via HDR. These point mutations altered the endogenous coding sequence from CAC→GCG, which changed the amino acid reside from histidine to alanine, H350A (red letters). An additional silent mutation (C→A) was included to add a restriction site for genotype analysis (red letter); (c) Schematic showing the mutated *Ido1* exon 10 locus encoding H350A in red.

Because of their normal growth and immune system development, constitutional IDOKO mice are widely used preclinically. However, this transgenic model does not reveal the role of IDO in different tissues or cell types. Recently, Bishnupuri et al. generated an intestinal epithelium specific IDO knockout mouse strain by breeding *Ido1*^tm1c(EUCOMM)Wtsi^ mice with the villin promoter *Cre* mice (105). The mouse colon cancer model utilizing this conditional IDO knockout mouse strain discovered neoplastic colon epithelium cell IDO to be a critical constituent of colon tumorigenesis and that Kyn metabolites rapidly activated PI3K-Akt signaling in the neoplastic epithelium to promote cellular proliferation and resistance to apoptosis. In addition to the constitutional and conditional IDO knockout mice that have previously been reported, our group has created new constructs that will allow for addressing novel questions including an IDO reporter mouse that expresses a IDO-GFP (Figure 2A), an IDO knock-in mouse under tissue-specific Cre using Rosa26Sor-loxp-IDO-FLAG-GFP (Figure 2B), and an IDO enzyme nullified mouse whereby the histidine of the 350th amino acid is substituted to an alanine (Figure 2C). We are currently characterizing each of these recently created models and will publish data supporting their phenotypes in the near future.

### The Expression, Immunosuppression, and Targetability of IDO in Subjects With GBM

Tumors arising from glia within the central nervous system (CNS) are considered to be potently immunosuppressive due to their surrounding immunospecialized neuroanatomical landscape (106). GBM is as an immunologically “cold” malignancy and associated with an immune system microenvironment that has resulted in the failure of all phase III clinical trials evaluating immunotherapy in patients with GBM to-date. Considering the work by our group and others in repeatedly demonstrating the remarkable pathogenic influence of IDO in subjects with GBM, the elucidation of its role and multi-variate functions may provide a path for enhancing the effectiveness of cancer immunotherapy against malignant glioma in the future.

IDO mRNA is highly expressed in ≥90% of GBM patients presenting with wild-type isocitrate dehydrogenase (wtIDH) and while not normally expressed, is inducible among a majority of human GBM cell lines after exposure to proinflammatory cytokines (23, 66, 107–110). While TDO expression is expressed at an even higher mRNA level than IDO in patient-resected GBM (33, 109–111), IDO2 levels are negligible or undetectable at the mRNA level (65), despite an IHC-focused study suggesting high IDO2 protein levels in GBM (*n* = 52) (109); the latter of which likely reflects conclusions based on non-specific antibody immunostaining. The cognate receptor for Kyn is AhR and is present throughout all grades of glioma, with the highest level reported in GBM. A recent study screening 75 glioma tissue samples by immunohistochemistry also confirmed higher IDO and TDO expression while low/no expression of IDO2 at protein levels (112). Studies from our group and others' have independently demonstrated that both Trp and Kyn are significantly decreased in GBM patient plasma as compared to human subjects without a tumor of the CNS (56, 57). Strikingly, in contrast to the absolute levels of metabolites, the Kyn/Trp ratio is significantly increased in GBM patient plasma long after surgical resection of the tumor which may reflect a treatment-related effect rather than due to the malignancy itself. A recent study by Kesarwani et al. provided additional insights into metabolomic profiling of newly diagnosed GBM (*n* = 80) and lower grade gliomas (LGG, *n* = 28) demonstrating that, GBM patients have increased intratumoral Trp and Kyn levels, but decreased KA as compared to LGG patients (110). Interestingly, Trp and Kyn accumulation was specific to classical and mesenchymal GBM subtypes, whereas KA accumulation was only evident in the proneural subtype. It's not clear why the systemic Kyn decreases while intratumoral Kyn increases in human subjects with GBM, which requires further investigation for both prognostic and therapeutic purposes.

Mechanistic studies investigating the full range of IDO-mediated immune modulation have primarily focused on the Kyn-AhR-Treg-MDSC (myeloid-derived suppressor cell) axis with data from several studies implying that alternative mechanisms of IDO behavior can occur. The inhibition of IDO enzyme activity in cultured human astroglioma cell lines decreases *de novo* synthesis of NAD^+^, which is associated with decreased tumor growth (113). The triple combination of IDO pathway inhibitor with chemo-radiation achieved better overall survival as compared to the dual combination of chemo-radiation (55). Interestingly, the survival benefit was abrogated in mice deficient for complement C3, potentially suggesting that IDO activity conveys immune evasion properties to tumor cells by suppressing complement activation (55). Finally, human GBM cell lines overexpress a translesion DNA polymerase, hpol κ, which helps to restore genome stability. Inhibition of AhR or the blockade of TDO enzyme activity decreases hpol κ expression, indicating that TDO contributes to tumor cell survival by supporting genome stability (114).

**Figure 3:**
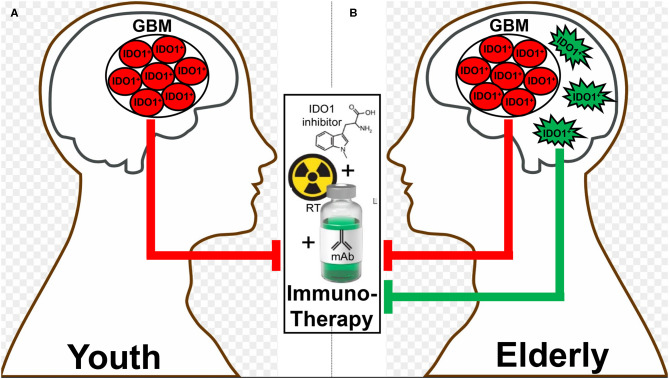
A hypothetic schematic for how advanced aging decreases immunotherapeutic efficacy in subjects with glioblastoma (GBM). There is a basal level of IDO-mediated immunosuppression by GBM cells (red) that does not change between **(A)** young and **(B)** elderly (as defined by ≥65 years of age) individuals. In contrast, there is additional immunosuppression in the elderly brain due to the accumulation of brain-resident IDO-expressing dendritic cells (green), which synergize with the GBM cell IDO to suppress the anti-GBM immune response facilitated by combination radiation, anti-PD-1 mAb, and IDO enzyme inhibitor treatment (middle box).

Different therapeutic interventions have profoundly different effects on both the systemic and local tumor-immune environment which include the alteration of IDO levels. In two recent glioma studies, both PCC0208009 (PCC), a potent IDO enzyme inhibitor with an IC_50_ of 4.52 nM, and RY103, an IDO-TDO dual inhibitor, have demonstrated suppression of tumor cell line- and intra-tumoral-IDO expression, respectively (112, 115). In contrary to the downregulation of IDO, a recent phase I clinical trial evaluating epidermal growth factor receptor variant III (EGFRvIII)-targeted chimeric antigen receptor (CAR) T cell therapy in GBM patients demonstrated a dramatic induction of intratumoral IDO expression after the adoptive transfer of CAR T cells (116). At almost the same time as when this clinical observation was reported, our group published a similar T cell-inducing IDO effect in humanized mice with intracranial human GBM and reconstituted with human immune cells (65). While the IDO-promoting effects of intratumoral T cell-infiltration appears to extend across human cancer types (3, 4), the association between IDO expression and overall survival depends on the type of tumor under investigation. This likely reflects the different composition of IDO expressing cells within tumors, the different function of intratumoral IDO that depends on the cell of origin, as well as for IDO expressing cells outside of the tumor microenvironment that also possess anti-cancer mechanisms depending on anatomical context. It remains notable that, to effectively target IDO with an enzyme inhibitor, the treatment must include an approach that yields robust inflammation such as irradiation. Accordingly, while studying the syngeneic GL261 in young C57BL/6 mice, we previously found that while neither radiation, anti-PD-1 mAb, nor IDO enzyme inhibitor treatment improved long-term survival as single- or dual-agent approaches, the simultaneous combination of all three modalities led to a remarkable synergistic durable survival improvement (87). Presumably, this was due to the induction of IDO by radiation, the neutralization of IDO enzyme activity by pharmacologic neutralization, and the enhancement of the anti-tumor immune response with PD-1 blockade. It's notable, however, that this triple combination was significantly less effective in older animal subjects when IDO expression is increased in the brain independent of tumor burden. As a means to explain this observation, we are currently investigating the working hypothesis that dendritic cells accumulate in the brain during advanced age (117, 118), express IDO and suppress immunotherapeutic efficacy (Figures 3A,B).

## Concluding Remarks

It has been >20 years since the initial study by Munn et al. that uncovered the immunosuppressive role of IDO (119). Although extensive studies have been conducted to elucidate the underlying mechanisms of IDO-mediated immunosuppression, our knowledge remains incomplete. Notwithstanding, a growing list of clinical trials aimed at inhibiting the immunosuppressive effects of IDO have been undertaken as single agent and combinatorial regimens (Table 1)—without any remarkable success stories to-date. To this end, there are questions in the field including those that we previously addressed (3), as well as new considerations such as: (i) what are the immunosuppressive contributions of enzyme- and non-enzyme-IDO activity; (ii) will an IDO neutralizing pharmacologic that degrades protein, rather than only inhibiting enzyme activity, provide a superior therapeutic effect; (iii) given that IDO is expressed in tumor-draining lymph nodes (29), within the tumor itself, and inside the brain parenchyma during advanced age, are all anatomical sites required for mitigation to achieve optimal immunotherapeutic efficacy; (iv) among the growing compendium of possible immunological modifiers (i.e., radiation, anti-CTLA-4 mAb, anti-PD-(L)1 mAb, etc.), what is the optimal immunotherapeutic cocktail for combining with an IDO pharmacologic inhibitor? Also, the functional similarity between IDO and TDO requires further study and the use of an IDO-TDO double knockout mouse model might be helpful in validating their potency and half-life. Future investigations should also include the rigorous assessment of expression levels combined with an *in vivo* analysis of Trp and Kyn for better understanding how this immunosuppressive mediator functions among tissues, treatments, and across the lifespan. Understanding the full immunobiology of IDO and the generation of better neutralizing agents will allow for the potential future achievement of applying optimal therapeutic effects in human subjects with malignant cancer(s).

**Table 1:** Ongoing and historical clinical trials that target IDO in cancer.

**Agent**	**Indication(s)**	**Phase**	**Status**	**Notes**	**NCT no**.
Indoximod (_D_-1-MT)	Metastatic solid tumor	I	Completed	Combined with docetaxel	NCT01191216
	Solid tumor	I	Completed	Single agent	NCT00567931
	Metastatic breast cancer	I/II	Active, not recruiting –> now completed	Combined with vaccine	NCT01042535
		II	Recruiting	Combined with fulvestrant or tamoxifen and palbociclib	NCT02913430
		II	Active, not recruiting –> now completed	Combined with docetaxel or paclitaxel	NCT01792050
	Melanoma	I/II	Recruiting –> now active, not recruiting	Combined with ipilimumab (CTLA-4 mAb), nivolumab, or. pembrolizumab	NCT02073123
	Metastatic Adenoma of Pancreas	I/II	Recruiting –> now completed	Combined with gemcitabine and nab-paclitaxel	NCT02077881
	Acute myeloid leukemia	I/II	Recruiting	Combined with cytarbine, idarubicin	NCT02835729
	GBM, glioma, gliosarcoma	I/II	Recruiting –> now active, not recruiting	Combined with temozolomide, bevacizumab (VEGF mAb) and radiation	NCT02052648
	GBM, glioma, ependymoma, medulloblastoma	I	Recruiting	Combined with temozolomide and radiation or cyclophosphamide and etoposide	NCT02502708
	Prostate carcinoma	II	Active, not recruiting–> now completed	Combined with sipuleucel-T	NCT01560923
	NSCLC	II	Recruiting –> now active, not recruiting	Combined with docetaxel and tergenpumatucel-L	NCT02460367
INCB024360	Advanced neoplasms	I	Completed	As single agent	NCT01195311
	Myelodysplastic Syndromes	II	Completed	As single agent	NCT01822691
	Melanoma	I/II	Recruiting –> now terminated	Combined with ipilimumab	NCT01604889
		II	Recruiting–> now completed	Combined with a multipeptide-based vaccine	NCT01961115
	Reproductive tract tumors	II	Completed –> now terminated	Compared to tamoxifen	NCT01685255
		I/II	Recruiting	Combined with vaccine and cyclophosphamide	NCT02785250
		I	Active, not recruiting	With therapeutic conventional surgery	NCT02042430
		I	Recruiting–> now completed	Combined with adoptive transfer of NK cells, IL-2, fludarabine, and cyclophosphamide	NCT02118285
		I/II	Recruiting–> now terminated	Combined with CRS-207 and Pembrolizumab (PD-1 mAb)	NCT02575807
		I/II	Recruiting	Combined with DC-targeted NY-ESO-1 and poly-ICLC	NCT02166905
		I/II	Withdrawn	As single agent	NCT01982487
		II	Suspended	Combined with pembrolizumab	NCT03602586
	Solid tumors	I/II	Recruiting–> now active, not recruiting	Combined with MK-3475	NCT02178722
		I	Recruiting–> now active, not recruiting	Alone or combined with combination of pembrolizumab, cisplatin, pemetrexed, carboplatin, or paclitaxel	NCT02862457
		I	Active, not recruiting	Combined with itacitinib (JAK inhibitor)	NCT02559492
		I/II	Recruiting–> now active, not recruiting	Combined with combination of pembrolizumab, ozaliplatin, leucovorin, 5-fluorouracil, gemcitabine, nab-paclitaxel, carboplatin, paclitaxel, pemetrexed, cyclophasphamide, or cisplatin	NCT03085914
		I/II	Recruiting –> now completed	Combined with MEDI4736 (PD-L1 mAb)	NCT02318277
		I/II	Active, not recruiting	Combined with azacitidine, pembrolizumab	NCT02959437
	Meta. colorectal cancer	I/II	Not yet recruiting	Combined with pembrolizumab and azacitidine	NCT03182894
	Gastric cancer	II	Not yet recruiting –> now recruiting	Combined with pembrolizumab	NCT03196232
	Meta. Pancreatic cancer	II	Not yet recruiting –> now recruiting	Combined with CRS-207, pembrolizumab, CY, CRS-207, GVAX	NCT03006302
		II	Withdrawn	Combined with pembrolizumab	NCT03432676
	^*^NSCLC, urothelial carcinoma	I	Active, not recruiting –> now terminated	Combined with atezolizumab (PD-L1 mAb)	NCT02298153
	SCCHN	II	Withdrawn	Combined with pembrolizumab	NCT03325465
	Head and neck cancer	III	Active, not recruiting	Combined with pembrolizumab vs. pembrolizumab alone or EXTREME regimen	NCT03358472
	Lung cancer	II	Active, not recruiting	Combined with pembrolizumab, platinum-based chemotherapy	NCT03322566
		II	Active, not recruiting	Combined with pembrolizumab	NCT03322540
	Renal cell carcinoma	III	Active, not recruiting	Combined with pembrolizumab vs. sunitinib and pazopanib	NCT03260894
	Muscle invasive bladder cancer	II	Not yet recruiting	Combined with pembrolizumab	NCT03832673
	Sarcoma	II	Recruiting	Combined with pembrolizumab	NCT03414229
GDC-0919	Solid tumors	I	Completed	As single agent	NCT02048709
(formerly NLG-919)	Locally-advanced or metastatic solid tumors	I	Active, not recruiting	Combined with MPDL3280A (PD-L1 mAb)	NCT02471846
IDO1 peptide	NSCLC	I	Completed	As single agent	NCT01219348
	Melanoma	I	Recruiting –> now completed	Combined with ipilimumab or vemurafenib (BRAF inhibitor)	NCT02077114
		II	Recruiting –> now terminated	Combined with temozolomide, imiquimod, GM-CSF, and survivin peptide	NCT01543464
PF-06840003	GBM or grade III anaplastic glioma	I	Recruiting –> now completed	As single agent	NCT02764151
BMS986205	Advanced cancer, melanoma, NSCLC	I/II	Recruiting	Combined with nivolumab and ipilimumab	NCT02658890
	Hepatocellular Carcinoma	I/II	Recruiting	Combined with nivolumab	NCT03695250
	Lip, oral cavity squamous cell carcinoma, pharynx, larynx, squamous cell carcinoma	II	Recruiting	Combined with BMS-986205	NCT03854032
	Advanced cancer	I	Recruiting –> now completed	Combined with nivolumab	NCT03192943
DN1406131	Advanced Solid Tumors	I	Not yet recruiting	As single agent	NCT03641794
HTI-1090^**^	Advanced Solid Tumors	I	Active, not recruiting	As single agent	NCT03208959
NLG802	Solid tumor	I	Active, not recruiting	As single agent	NCT03164603
SHR9146+SHR-1210	Solid tumor, metastatic cancer, neoplasm malignant	I	Not yet recruiting	Combined with apatinib	NCT03491631
MK-7162	Solid Neoplasm	I	Recruiting	Combined with pembrolizumab	NCT03364049

* *NSCLC, non-small cell lung cancer; DLBCL, diffuse large B-cell lymphoma; SCCHN, squamous cell carcinoma of head/neck; UC, urothelial carcinoma*.

** *IDO1-TDO dual enzymatic inhibitor*.

## Author Contributions

LZ and DW generated a draft of the manuscript. AB, EL, KL, LB, JS, BZ, JW, SM, JM, RL, EW, LD, GS, and RM provided feedback on the draft and/or data for inclusion. All authors contributed to the article and approved the submitted version.

## Conflict of Interest

The authors declare that the research was conducted in the absence of any commercial or financial relationships that could be construed as a potential conflict of interest.
